# Early life adverse experiences and loneliness among young adults: The mediating role of social processes

**DOI:** 10.3389/fpsyg.2022.968383

**Published:** 2022-09-20

**Authors:** Jyllenna Landry, Ajani Asokumar, Carly Crump, Hymie Anisman, Kimberly Matheson

**Affiliations:** ^1^Department of Neuroscience, Carleton University, Ottawa, ON, Canada; ^2^The Royal’s Institute of Mental Health Research, University of Ottawa, Ottawa, ON, Canada

**Keywords:** loneliness, early life trauma, emotional abuse, social support, young adult

## Abstract

Loneliness has been described as endemic among young people. Such feelings of social isolation ‘even in a crowd’ are likely linked to adverse early life experiences that serve to diminish perceptions of social support and intensify negative social interactions. It was suggested in the present series of survey studies that childhood abuse, which compromises a child’s sense of safety in relationships, may affect social processes that contribute to loneliness in young adulthood. Study 1 assessed different adverse childhood and adult experiences in relation to loneliness among young adults (*N* = 171). Linear regression analyses indicated that childhood abuse was uniquely associated with greater loneliness, and this relationship was partially mediated by the perceived availability of social support. Study 2 (*N* = 289) assessed different forms of childhood abuse and demonstrated that early life emotional abuse was a unique predictor of loneliness, and this relationship was fully mediated by lower perceived support or value in social connections (social connectedness) and more frequent unsupportive interactions with friends. Study 3 evaluated the implications of the age of occurrence of abuse (*N* = 566). Both emotional and sexual abuse predicted young adult loneliness regardless of age; abuse that was recalled to have occurred at very early ages (0–5 years) was not predictive of loneliness over and above consideration of events that happened in older childhood. These relationships were at least partially mediated by perceived social support, social connectedness, and in the case of emotional abuse, unsupportive interactions with friends. Our results add to mounting evidence pointing to the prevalence of loneliness among young adults and the links to adverse early life experiences that may serve to shape appraisals of safety, value, and personal worth in social relationships.

## Introduction

The prevalence of loneliness and its association with adverse health outcomes has become increasingly salient to the general public and among health professionals. Loneliness has been described as an epidemic (Jeste et al., 2020) and pandemic (Palgi et al., 2020). The United Kingdom and Japan each recently appointed a Minister for Loneliness (Basu, 2021). Most recently, the COVID-19 pandemic rendered the consequences of loneliness highly visible, including associations with greater occurrence of anxiety, depression, and elevated substance use (Meade, 2021; Knox et al., 2022). These links were especially marked among younger individuals (Varma et al., 2021), including university students (Bu et al., 2020) who are increasingly being recognized as a group at risk (Diehl et al., 2018; Hysing et al., 2020).

While much attention was paid to these issues through the COVID-19 pandemic, the prevalence of loneliness was not unique to the global social distancing policies that were invoked. Even prior to COVID-19, young adults (18–22 years) were described as the ‘loneliest generation of Americans’ (Cigna, 2018) with 20–48% reporting severe levels of loneliness (Williams and Braun, 2019). Loneliness at this stage of life poses developmental risks as the young adult years are marked by several transitions, including the expansion of their social world beyond the family, identity exploration, and greater autonomy (Kirwan et al., 2021). Thus, loneliness may be problematic for many adolescents and young adults, being related to cognitive and physical maturation, as well as developmental changes in social autonomy, perspective-taking, and individuation (Laursen and Hartl, 2013; Buecker et al., 2021).

Loneliness does not merely encompass social isolation but includes the psychological torment and pain that comes from a lack of meaningful relationships. Thus, loneliness is both a relational experience and an emotional one (Hawkley and Cacioppo, 2010; Von Soest et al., 2020). The factors that promote or prevent loneliness likely have strong roots in opportunities and safe environments that enable individuals to take full advantage of their social relationships in a manner that ensures well-being. Conversely, encounters that undermine trust, emotional connections to others, or that have been fraught with personal violation or emotional betrayal might diminish the ability to derive socially meaningful relationships. In this regard, early life socio-emotional events may play a significant role in whether individuals experience loneliness as young adults. It was the goal of the present investigation to assess the relations among adverse early life experiences, young adults’ social experiences and perceptions, and their reports of loneliness.

### Childhood experiences and loneliness

Both childhood and adult trauma experiences were predictive of feelings of loneliness among a range of populations (Kearney et al., 2018; Hyland et al., 2019). The disposition to loneliness and the trajectory toward loneliness among young people is variable over time and across individuals, being related to differences in emotional stability, agreeableness, and extraversion (Vanhalst et al., 2013). Of the many factors that can proactively influence adult well-being, adverse early life experiences may have especially pronounced consequences (Hays-Grudo and Morris, 2020), including links to anxiety and depressive disorders, and suicidal ideation (Anda et al., 2006; Brown et al., 2009; Hays-Grudo and Morris, 2020). It has been reported that childhood abuse was associated with elevated loneliness among young adults (Flett et al., 2016; Arslan and Yıldırım, 2021), and loneliness might represent a mediating factor in the relationship between abuse and psychological disorders (Shevlin et al., 2015). Among other things, traumatic childhood experiences may give rise to feelings of social indifference together with loneliness, which was predictive of suicidal ideation. However, the strength of these relationships diminished when individuals were recognized by others in relevant social groups (Wang et al., 2022).

Adverse childhood experiences may take many forms that can reflect environmental factors (e.g., living in poverty or unsafe neighborhoods) and relational factors, ranging from emotional, physical, or sexual abuse to disturbed parent-child interactions (e.g., neglect, disengagement from children, as well as hostility and coercion) (Rebbe et al., 2017). Some of these experiences may reflect multiple concurrent elements of a particular early life environment (e.g., parental substance use may co-occur with child neglect), and might represent the cumulative occurrence of events over time. While a greater number of adverse childhood events is associated with poorer adult health and wellness outcomes (Hughes et al., 2017; Petruccelli et al., 2019), the nature of these early life experiences may elicit different processes and outcomes (Rebbe et al., 2017; Shin et al., 2018). For example, both childhood physical and emotional abuse were related to suicidal ideation through their links to anxiety, whereas childhood neglect was tied to suicidal ideation through diminished social support (Bahk et al., 2017). Other researchers using latent class analysis of adverse childhood experiences have found commonalities in patterns associated with experiences involving deprivation or violence (Henry, 2020). However, the psychosocial mechanisms linking varying types of adverse experiences with mental health outcomes are less well understood, although multiple biopsychosocial processes are likely implicated (Anisman et al., 2018; Barrero-Castillero et al., 2022).

Young children are typically incapable of making accurate appraisals of situations and may misinterpret parental mistreatment and form inappropriate inferential attributions for their experiences to aspects of themselves, thereby promoting self-blame and diminished self-esteem (Hays-Grudo and Morris, 2020). Coupled with misappraisals, young children may lack effective coping strategies that might otherwise diminish distress (Compas et al., 2017). Cumulative adverse experiences may come to undermine school performance, disturb the ability to form and maintain close relationships, foster mistrust of others, and impair self-regulation, all of which may favor the emergence of psychological disorders in young adulthood (Kisely et al., 2018; Fitzgerald and Gallus, 2020; Karatekin and Ahluwalia, 2020; Colburn et al., 2021). Of course, the impact of early life experiences on the development of loneliness can be influenced by a gamut of psychosocial factors, including age and gender, maladaptive cognitive schemas, epigenetic factors, and socioeconomic status, among many others (Southwick et al., 2014).

### Diminished perceptions of social support

Irrespective of age, several social factors, including number of friends, social engagement, and frequency of contact have been tied to feelings of loneliness (Luhmann and Hawkley, 2016). Not surprisingly, such social assets have been identified as critical protective factors that promote resilience in the face of adverse childhood experiences (Sperry and Widom, 2013; Fitzgerald and Gallus, 2020; Leung et al., 2022). Conversely, social ostracism partially mediated the link between adverse childhood experiences and later feelings of loneliness (Arslan and Yıldırım, 2021). Young adults who had experienced more adverse events in their household while growing up perceived less social support (Gayman et al., 2011; Caravaca-Sánchez et al., 2019; Karatekin and Ahluwalia, 2020; Colburn et al., 2021), which predicted greater symptoms of anxiety and depression (Watt et al., 2020). Likewise, childhood physical and emotional abuse were associated with diminished social networks and greater perceived peer rejection in adulthood, which were linked to greater loneliness (Gibson and Hartshorne, 1996; Lev-Wiesel and Sternberg, 2012). It has been suggested that the shame associated with abusive experiences may promote negative perceptions of social support, and hence decreased disclosure of traumatic events (Aakvaag et al., 2019), which might undermine the longer-term capacity to cope effectively with such experiences.

Many adverse childhood experiences are inherently relational, often being committed by an individual who is an important attachment figure and should be a protective influence for the child. This may foster maladaptive cognitive processing of emotionally intense situations and limitations in emotional regulation and social skills (Dvir et al., 2014; Gama et al., 2021). In addition, the experience of childhood abuse may have important implications for how an individual appraises social support and social connection (Williams and Galliher, 2006; Dodson and Beck, 2017). Specifically, an individual may determine that the risks involved with social connections are not worth the potential benefits and may not actively seek out or appreciate the social support that is available to them (Lee and Robbins, 1995; Lee et al., 2001; Williams and Galliher, 2006), choosing social isolation instead of connection (Arslan and Yıldırım, 2021). The interpersonal foundation of childhood abuse and maltreatment has been proposed as a cogent mechanism by which child abuse promotes later life psychological distress and mental health challenges, including loneliness (Fitzgerald and Gallus, 2020).

### The present investigation

Early life adverse experiences can profoundly influence psychosocial functioning among young adults. Yet, little is understood about the socio-emotional wellness of young people with respect to their feelings of loneliness, despite its prevalence and documented connection to mental health. A cluster of early life factors has been linked to loneliness among young adults, including reports of early life trauma, household adversity, psychological maltreatment, and various forms of abuse. There is emerging evidence that different forms of early life adversity trigger varying psychological trajectories. Similarly, diverse social processes have been implicated in the relations between childhood experiences and loneliness, including perceived social support, social isolation, social skills, rejection, and recognition or ostracism from others. Thus, the present multi-study investigation, conducted prior to the COVID-19 pandemic, explored various early life adverse experiences and their relation to loneliness, together with several aspects of social functioning to better understand mechanisms that link the childhood experiences and loneliness in young adulthood. In Study 1, we assessed different forms of trauma experiences in relation to loneliness among young adults, including the mediating role of perceived social support from parents and peers. Study 2 further assessed relations between different forms of childhood abuse and loneliness and expanded on potential social mediators including social connectedness and unsupportive social interactions with parents and peers. Finally, Study 3 assessed whether the age at which specific forms of abuse were experienced had differential implications for social processes and the relationships with loneliness.

## Study 1

While adverse childhood experiences have been found to be predictive of loneliness and the emergence of other psychopathologies in adulthood, what is rarely taken into consideration is the proliferation of stressors that may co-occur with early life adversities. In this regard, adverse childhood experiences have been associated with an elevated risk of subsequent stressor encounters (Widom et al., 2008; Radford et al., 2013). In addition, re-victimization is common among childhood abuse survivors, and it is possible that these later experiences could account for adult wellness (Grasso et al., 2016; Goemans et al., 2021). In Study 1, the relations between exposure to a range of traumatic events (including experiences of adult victimization) and loneliness in young adulthood were assessed. Of particular interest was whether abusive childhood experiences uniquely predicted loneliness relative to other forms of trauma that may be encountered either in adulthood or childhood.

While multiple aspects of social relationships may be affected by early life experiences, a lack of perceived social support is a strong predictor of loneliness (Wang et al., 2018). Social support has been shown to mediate the relationship between experiences of early life trauma and abuse and loneliness in adolescence and adulthood (Runtz and Schallow, 1997; Sperry and Widom, 2013; Watt et al., 2020). The differential effects of such support from friends relative to parents may be especially pertinent during the transition to young adulthood (Riggio et al., 1993; Chen and Feeley, 2014). Adolescence is a developmental stage in which individuals’ identity begins to move from parental influences to those provided by peers, and the social-emotional skills that are critical to establishing supportive peer relationships are shaped by individuals’ well-being and sense of self (Mitic et al., 2021). It was hypothesized that the relationship between abusive childhood experiences and current loneliness would be mediated by diminished perceptions of social support. As the comparative effects of various sources of perceived support (i.e., parents, friends) in relation to loneliness are not yet well understood (Fitzgerald and Gallus, 2020), the relative contribution of perceived support from friends or parents was also evaluated.

### Materials and methods

#### Participants and procedures

Participants were first-year undergraduate students aged 25 years or less recruited online through a computer registry (SONA system), and comprised primarily white/Caucasian females (see Table 1). Upon provision of informed consent, participants completed a series of measures, after which they were fully debriefed, and provided with course credit for their participation and contact information should they experience any distress. The study protocol was approved by the Research Ethics Board at Carleton University (REB#: 03-006).

**TABLE 1:** Demographic description and prevalence of loneliness for participants of all three studies.

		Study 1 (*N* = 171)		Study 2 (*N* = 289)		Study 3 (*N* = 566)	
		*n*	%	*n*	%	*n*	%
Gender	Male	54	31.6	75	26.0	147	26.1
	Female	117	68.4	214	74.0	416	73.9
Ethnoracial identity	White/Caucasian	111	72.5	185	64.0	319	56.6
	Asian	26	17.1	66	22.9	146	25.9
	Black	9	5.9	24	8.3	62	11.0
	Indigenous	2	1.3	5	1.7	5	0.9
	Mixed/Other	5	3.3	9	3.1	32	5.7
Age (years)	Mean/*SD*	19.10	1.52	18.76	1.59	19.11	1.69
Loneliness	Score < 34	59	34.5	75	26.0	140	24.7
	Score 35 – 49	68	39.8	137	47.4	247	43.6
	Score ≥ 50	44	25.7	77	26.6	179	31.6

Missing not included in calculations; missing < 1% except for ethnoracial identity in Study 1 (missing *n* = 18, 10.5%).

#### Measures

##### Demographics

Participants were asked to identify their gender, age, and ethnoracial background using an open-ended question format.

##### Loneliness

The UCLA Loneliness Scale Version 3 (Russell, 1996) includes 20 items that assess perceived loneliness (e.g., “How often do you feel alone?”), social behaviors (e.g., “How often do you feel shy?”), and quality of relationships (e.g., How often do you feel that your relationships with others are not meaningful?”). Respondents rated the frequency of such feelings from 1 (never) to 4 (always) and ratings across the items were summed (Cronbach’s α = 0.89) to provide scores with a possible range of 20–80.

##### Social support provisions

Perceptions of social support provided by parents and friends were assessed using Cutrona and Russell’s (1987) Social Provisions Scale comprising 12 items in relation to each of the sources of support. Participants indicated whether a range of supportive behaviors was provided by each of the sources on a 3-point rating scale: no (1), not sure (2), and yes (3). Mean scores were calculated for the social support provided by parents (Cronbach’s α = 0.89) and friends (Cronbach’s α = 0.89). Perceptions of support from these sources was moderately correlated, *r* = 0.40, *p* < 0.001.

##### Trauma experiences

The Traumatic Life Events Questionnaire (TLEQ) (Kubany et al., 2000) identifies significant traumatic life events at various points across the participant’s life. This measure comprises a broad spectrum of potentially traumatic events, ranging from natural disasters, accidents, and assaults, to childhood abuse. Events are described in behaviorally descriptive terms. The frequency of occurrence of each event is assessed using a 7-point scale on which participants indicate whether each event has occurred from never (0) to more than five times (6). For the present study, our interest in various trauma exposures resulted in consideration of five experiences, including (1) non-social experiences of shock (e.g., being in a car accident), social experiences of (2) loss (i.e., the death of a loved one) or (3) having something bad happen to a loved one (e.g., witnessing assault), (4) traumas that involved social threats to the participant directly as an adult (e.g., physically hurt by an intimate partner or threatened by a stranger), and (5) abuse ‘while growing up’ (i.e., physical punishment and inappropriate sexual interactions) (based on Breslau et al., 1999).

Trauma exposure was calculated in two ways. To assess whether different types of trauma were differentially associated with loneliness, for each of the five trauma types, the average occurrence of the respective events was calculated irrespective of the age at which they were experienced. Second, early life trauma exposure was calculated by counting the number of events participants reported as having occurred 10 or more years ago (given the mean age of the participants, events that happened 10 or more years ago likely constituted childhood experiences) collapsed across the trauma types (with the exception of abuse while growing up).

#### Statistical analyses

Frequencies of traumatic events were reported, and gender differences were assessed using independent *t*-tests. Zero-order relationships among variables were explored using Pearson correlations. To determine whether different forms of trauma were uniquely predictive of loneliness, two linear regression analyses were conducted wherein the five trauma types (at any age; or those experienced 10 or more years ago) were entered simultaneously as predictors. A mediation analysis assessed whether the relations between childhood abuse and loneliness could be accounted for by the diminished levels of perceived social support from friends and parents. The PROCESS macro applying model 4 (Hayes, 2022) was used with bootstrapping procedures using 5,000 resamples to establish the 95% confidence intervals (CI) to assess significance. A follow-up analysis was conducted to determine whether gender moderated the mediated model (PROCESS model 8). For each analysis, the power to detect a medium effect size of partial *R*^2^ = 0.05 at *p* = 0.05 with the sample size of the present study was β = 0.85.

### Results

The most common form of traumatic event reported by participants was the death of a loved one (*n* = 94, 55.0%). However, other events were not uncommon, including those constituting a severe shock (*n* = 54, 31.6%), witnessing negative experiences among loved ones (*n* = 75, 43.9%), or even personal assault as an adult (*n* = 44, 25.7%). While the majority of participants did not report experiencing any of these forms of trauma as children (i.e., more than 10 years ago), 19.3% (*n* = 33) reported at least one early life general trauma experience. In addition, 25.7% (*n* = 44) indicated some form of childhood physical or sexual abuse. There was a significant relationship between experiencing abuse as a child and assault in adulthood, *r* = 0.37, *p* < 0.001. There were no gender differences in trauma experiences, nor in feelings of loneliness (*M* = 42.35, *SD* = 12.67). As seen in Table 1, about a third of participants reported relatively low levels of loneliness (scores of 34 or less), but a quarter had moderately high to high scores (50 or greater).

A multiple linear regression analysis conducted with the five trauma types entered together as predictors indicated that, as a whole, traumatic events were not associated with greater loneliness, *R*^2^ = 0.054, *F*(5,164) = 1.86, *p* = 0.104. However, examination of the regression coefficients indicated that reports of childhood abuse were uniquely associated with greater loneliness, *b* = 2.82, *SE* = 1.16, *p* = 0.016, *r* = 0.22, *p* = 0.002. Assault as an adult was correlated with greater loneliness, *r* = 0.13, *p* = 0.052, whereas other trauma types were not significantly related to loneliness (*p*s > 0.17).

The multiple regression analysis with traumas experienced early in life trauma (more than 10 years ago) as predictors showed that early life experiences were associated with greater loneliness, *R*^2^ = 0.064, *F*(2,168) = 5.07, *p* = 0.004. Examination of the regression coefficients indicated that, once again, only reports of childhood abuse were associated with greater loneliness, *b* = 6.10, *SE* = 1.84, *p* = 0.001, whereas other early life traumas were not, *r* = 0.05, *b* = 1.23, *SE* = 2.10, *p* = 0.559.

**FIGURE 1:**
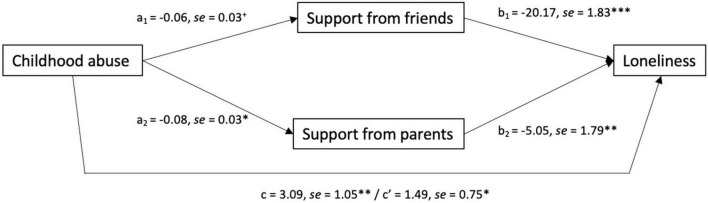
Mediated relationship between experiences of childhood abuse and loneliness through perceived social support from friends and parents in Study 1. Path coefficients indicate that childhood abuse was associated with perceived support and such perceptions were, in turn, related to loneliness. Perceived support partially accounted for the relationship between experiences of child abuse and loneliness among young adults. c represents the total effect, whereas c’ represents the direct effect. ^+^*p* < 0.10; *p < 0.05; ^**^p < 0.01; ^***^p < 0.001. *N* = 171.

As childhood abuse was the only trauma type that was uniquely associated with loneliness, the extent to which this relation was mediated by diminished levels of perceived social support from parents or friends was assessed. As seen in Figure 1, the relationship between childhood abuse and loneliness was partially accounted for by lower perceived social support, Effect = 1.60, CI_0.95_[0.02, 3.65], but not uniquely from friends, *a_1_b_1_* = 1.21, CI_0.95_[–0.18, 3.04], or parents, *a_2_b_2_* = 0.40, CI_0.95_[–0.03, 1.07]. Gender did not significantly moderate the mediated relationships.

### Discussion

As identified in previous research (Cigna, 2018; Williams and Braun, 2019), high levels of loneliness were prevalent in over a quarter of this population of young adults. In addition, a sizable proportion had experienced some form of traumatic event, and in particular, childhood abuse in the form of excessive physical punishment or inappropriate non-consensual sexual interactions was reported by more than 1 in 4 young adults. Although other types of childhood or adult traumatic experiences were common, they were not predictive of loneliness, whereas childhood abuse was related to increased loneliness as a young adult. It seems the social threat emanating from childhood abuse may be an important mechanism associated with later life well-being, whereas experiences reflecting more general adverse childhood experiences or the effects of a broad range of stressors encountered as young adults were less prominent. Childhood abuse was associated with a higher likelihood of revictimization in the form of assault in young adult years. Although adult assault experiences were related to loneliness, they did not account for unique variation when childhood abuse was controlled. This would suggest that the early life experiences play a greater role than those that might be reflective of adult revictimization in shaping feelings of loneliness among young adults.

The relation between childhood abuse and loneliness was partially mediated by lower perceptions of social support, which aligns with research demonstrating the mediating role of social support in the relationship between childhood abuse and well-being more generally (Runtz and Schallow, 1997; Fitzgerald and Gallus, 2020; Watt et al., 2020). As in earlier research, individuals who experienced childhood abuse were more likely to perceive lower social support from both parents and peers (friends) (Gayman et al., 2011; Caravaca-Sánchez et al., 2019; Karatekin and Ahluwalia, 2020; Colburn et al., 2021). Thus, the experience of childhood abuse may have important implications for how an individual appraises the availability of social support across sources, and may have significant implications for their ability to fill social needs (Gayman et al., 2011; Flett et al., 2016; Von Soest et al., 2020).

An important limitation of Study 1 was the assessment only of childhood sexual or physical abuse. Notably absent were experiences of emotional abuse. This is particularly relevant given that various forms of childhood abuse (i.e., sexual abuse, physical abuse, or emotional abuse) can have differential implications for later life mental health (Kisely et al., 2018; Poole et al., 2018; Colburn et al., 2021; Gama et al., 2021), as well as for social support resources.

## Study 2

The numerous socio-emotional outcomes of childhood abuse, including shame and poor views of the self (Wright et al., 2009), a belief that one does not matter to others (Flett et al., 2016), lack of trust in others (Dodson and Beck, 2017), and low social skills (Li et al., 2022) can result in an individual feeling low social connectedness (Lee et al., 2001). Social connectedness is a relational schema that goes beyond perceptions of social support to encompass the value an individual places on their relationships with others and their sense of belonging (Lee and Robbins, 1998; Lee et al., 2001). Importantly, because the cognitive schemas that shape social connectedness begin to form in early life, they may be particularly prone to adverse childhood experiences that shape one’s view of the self in social situations (Lee et al., 2001; Wright et al., 2009). Low social connectedness has been proposed to be a coping mechanism designed to protect an individual from further harm to the self that others may inflict (i.e., rejection), and hence limits perceptions of social support (Lee et al., 2001) and may exacerbate feelings of loneliness.

Possibly due to trust and communication issues, an individual may not only be less likely to perceive that support is available, but might encounter more unsupportive responses from friends or parents when help is sought (Poole et al., 2018; Alink et al., 2019). Childhood abuse may influence choices in adult relationships that compound the likelihood of negative social interactions (Fergusson and Horwood, 1999; Aakvaag et al., 2019), either because they have been exposed to predominantly unsupportive relationships as children (Gayman et al., 2011; Von Soest et al., 2020) or because poor social functioning limits their friendship options (Lev-Wiesel and Sternberg, 2012; Li et al., 2022).

Study 2 broadened our assessment of childhood abuse and considered the mediating role of multiple aspects of social relationships. As Study 1 did not demonstrate that perceptions of support from friends relative to parents were differentially associated with reported child abuse or loneliness, Study 2 assessed perceived support in general, together with an overall sense of social connectedness. However, cognizant of the importance of establishing positive peer relationships through the transition to adulthood and the potentially powerful implications of not acquiring support from friends though this period, we evaluated the mediating role of unsupportive interactions with each of friends and parents in the relationship between trauma and loneliness in young adults.

### Materials and methods

#### Participants and procedure

As in Study 1, participants (*N* = 289) were first-year undergraduate students aged 25 years or less, recruited online through a computer registry (SONA system), and comprised a primarily female and White/Caucasian sample (see Table 1). Upon provision of informed consent, as in Study 1, participants provided demographic information and completed the Traumatic Life Events Questionnaire and the UCLA Loneliness Scale (Cronbach’s α = 0.89), along with additional measures of early life abuse and indices of social support. The study protocol was approved by the Research Ethics Board at Carleton University (REB#105169).

#### Additional measures

##### Early Life Trauma Inventory (ELTI)

This measure of trauma assessed the self-reported occurrence of traumatic events occurring before the age of 18 years (Bremner et al., 2007). Respondents rated the frequency of 27 events from 0 (never) to 5 (more than 10 times). The events reflected four types of trauma exposure, namely (1) general trauma (e.g., natural disaster, death, serious accidents, violence) (Cronbach’s α = 0.94); (2) physical punishment (i.e., physical contact or restraint with the purpose of causing physical injury to the victim) (Cronbach’s α = 0.89); (3) emotional abuse (i.e., verbal harm in the form of shameful and demeaning communication targeted to the victim) (Cronbach’s α = 0.94); and (4) sexual events (i.e., unwanted sexual contact that satisfies the perpetrator and/or humiliates the victim) (Cronbach’s α = 0.86).

##### Social support perceptions

As a distinction between support from parents and peers was not evident in Study 1, Study 2 employed a more comprehensive measure of perceived support from others in general (Cutrona and Russell, 1987). This version of the Social Provisions Scale comprised 24-items that assessed the degree of support participants perceived in their current relationships, rated on a scale from 1 (strongly agree) to 4 (strongly disagree) and averaged across all items (Cronbach’s α = 0.92).

##### Social connectedness

This measure comprised 20 items that assessed individuals’ sense of belonging and connection within their social world (e.g., “I find myself actively involved in people’s lives”; “Even around people I know, I don’t feel that I really belong” – reverse-scored) (Lee et al., 2001). Respondents rated each statement from 1 (strongly disagree) to 6 (strongly agree), and ratings were averaged to reflect greater social connectedness (Cronbach’s α = 0.93).

##### Unsupportive social interactions inventory

Unsupportive interactions with friends and parents were assessed in terms of 24 items that assessed various unsupportive responses, including distancing (e.g., “Would change the subject before I wanted to”), bumbling (e.g., “Would not seem to know what to say, or would seem afraid of saying or doing the “wrong” thing”), minimizing (e.g., “Would try to cheer me up when I was not ready to”), and blaming (e.g., “Would ask “why” questions about my role in the event.”) (Ingram et al., 2001). Respondents were first asked to think about times they turned to their friends for support before rating their experiences from 0 (none) to 4 (a lot) (Cronbach’s α = 0.93) followed by responding to their interactions with their parents (Cronbach’s α = 0.93). In both instances, average scores across the items were calculated to reflect greater unsupportive interactions.

#### Statistical analyses

The same approach to statistical analyses followed in Study 1 was applied in Study 2. The power to detect a medium effect size of partial *R*^2^ = 0.05 at *p* = 0.05 with the number of variables and sample size of the present study was β = 0.97. For none of the variables did missing data exceed 1%.

### Results

Based on responses to the Early Life Trauma Inventory, males were more likely to report early life experiences of physical punishment (*M* = 1.99, *SD* = 1.86) than females (*M* = 1.19, *SD* = 1.47), *F*(1,286) = 14.05, *p* < 0.001, whereas females were more likely to report inappropriate sexual encounters (*M* = 0.75, *SD* = 1.45) than males (*M* = 0.27, *SD* = 0.76), *F*(1,286) = 7.50, *p* = 0.007. There were no gender differences in reports of emotional abuse (*M* = 1.35, *SD* = 1.71) or general trauma exposure (*M* = 1.90, *SD* = 1.66). Correlations among adverse experiences were all moderately positive, ranging from *r* = 0.20 (physical punishment and sexual events) to *r* = 0.46 (between physical punishment and emotional abuse). Notably, general trauma exposure reported in responses to the TLEQ was not associated with any of the dimensions of early life trauma assessed using the ELTI, whereas childhood abuse reported on the TLEQ was associated with higher reports of physical punishment, *r* = 0.34, *p* < 0.001, sexual events, *r* = 0.48, *p* < 0.001, and emotional abuse, *r* = 0.30, *p* < 0.001, along with traumas in general, *r* = 0.27, *p* < 0.001.

There was no gender difference in feelings of loneliness (*M* = 43.01, *SD* = 11.40). As in Study 1, about a quarter of the sample expressed moderately high to high loneliness scores (50 or greater) (Table 1).

The multiple regression analysis assessing the relations between experiences of early life trauma (from the ELTI) and current loneliness was significant, *R*^2^ = 0.165, *F*(4,282) = 13.98, *p* < 0.001. Examination of the regression coefficients in Table 2 indicated that only reports of emotional abuse were uniquely associated with greater loneliness. While the other forms of trauma were mildly correlated with loneliness, none accounted for unique variance.

**TABLE 2:** Linear regression coefficients predicting loneliness from early life experiences of trauma assessed using the ELTI in Study 2.

	*b*	*SE*	*B*	*r*
Physical punishment	–0.81	0.44	–0.11	0.10*
Sexual events	0.37	0.50	0.04	0.16**
Emotional abuse	2.81	0.44	0.42***	0.39***
General trauma	0.20	0.41	0.03	0.17**

*p < 0.05; **p < 0.01; ***p < 0.001. *N* = 287.

**FIGURE 2:**
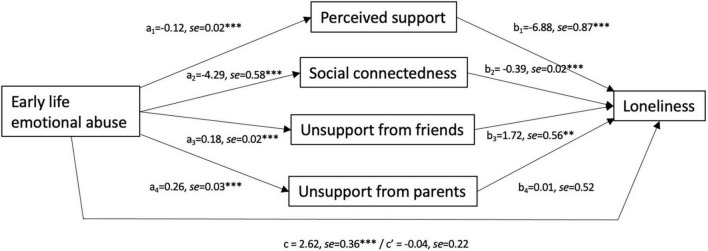
Mediated relationship between experiences of early life emotional abuse and loneliness through social support characteristics in Study 2. Path coefficients indicate that emotional abuse was associated with perceptions of support, social connectedness and unsupport from both friends and parents, and all but unsupportive interactions with parents predicted, in turn, feelings of loneliness. Although emotional abuse was related to loneliness, the diminished social processes fully mediated this relationship. c represents the total effect, whereas c’ represents the direct effect. ^**^p < 0.01; ^***^p < 0.001. *N* = 287.

A mediation analysis assessed whether the relation between emotional abuse and loneliness could be accounted for by the diminished levels of social support experienced (lower perceived support, social connection and more unsupportive interactions with friends and parents). As seen in Figure 2, the relation between early life emotional abuse and loneliness was fully accounted for by social processes, including diminished perceptions of social support, *a_1_b_1_* = 0.70, CI_0.95_[0.43, 1.01], lower social connectedness, *a_2_b_2_* = 1.66, CI_0.95_[1.24, 2.12], and more unsupportive interactions with friends, *a_3_b_3_* = 0.30, CI_0.95_[0.08, 0.54]. While emotional abuse was related to reports of more unsupportive interactions with parents, these reports were not associated with loneliness, and did not contribute to mediating the relationship between emotional abuse and loneliness, *a_4_b_4_* = 0.00, CI_0.95_[–0.29, 0.30]. A follow-up analysis to determine whether gender moderated the mediated model indicated that gender did not significantly moderate any of the mediated relationships.

### Discussion

As in Study 1, early life adverse experiences were associated with feelings of loneliness in young adults. Although the range of experiences was related to adult loneliness, only emotional abuse was a unique predictor. This aligns with previous research suggesting that loneliness in adolescents and emerging adults is not simply a relational issue but may be indicative of socio-emotional disturbances (Flett et al., 2016; Arslan and Yıldırım, 2021). In this regard, emotional abuse was further associated with diminished support perceptions no matter which index of support was considered. In turn, the link between emotional abuse and loneliness was fully mediated by diminished support perceptions and social connectedness and more unsupportive encounters with friends. Emotional abuse may play a role in shaping cognitive appraisals of safety, value, and personal worth in relationships (Lee et al., 2001; Wright et al., 2009; Flett et al., 2016). In addition, such abuse might decrease the capacity to elicit effective social support from peers (Poole et al., 2018), or may render the individual more likely to seek friendships with deviant or abusive peers (Lev-Wiesel and Sternberg, 2012; Gama et al., 2021). Any of these factors may place young adults at risk for loneliness as they navigate through new social contexts outside of the familial home. In contrast, unsupportive interactions with parents did not appear to be associated with loneliness. It is possible that if parents were perpetrators of emotional abuse, such encounters are anticipated, and may be less directly influential in relation to other social experiences.

All of the types of abuse evaluated by the measure of early life trauma (ELTI) used in Study 2 were related to recollections of childhood abuse while growing up based on the Traumatic Life Events Questionnaire. These relations suggest that the TLEQ and ELTI tapped into common recollections of early life abuse. However, as ELTI responses reflected any events prior to the age of 18, for university-aged young people such reports conflate recent experiences with those that occurred in childhood. The age at which trauma experiences occur is an important variable influencing the mental health implications among young adults (Khan et al., 2015; Grasso et al., 2016; Schalinski et al., 2016). Thus, a limitation of this measure is the inability to differentiate early life events from the recent experiences of young adults.

## Study 3

While prospective studies are ideal for assessing developmental trajectories and causal relations, prospective analyses of childhood abuse are fundamentally difficult, to say nothing of being ethically challenging to conduct. Studies using retrospective recall have inherent limitations, including biases stemming from more recent experiences. Despite recall biases, recollections of early trauma can play a meaningful role in predicting trauma in later childhood and adolescence (Grasso et al., 2016), as well as providing an understanding of how those experiences are appraised and how individuals cope (Wright et al., 2009). Moreover, it may be when the individual reaches adulthood that they are able to reflect clearly on the impact of such early experiences (Baker, 2009). In this regard, a child may not have the capacity to understand that certain behavior emanating from their caregiver constitutes abuse or have the ability to articulate this. Thus, while recent experiences may bias recall of early life traumas, emerging adulthood may provide the first opportunity for the victim of childhood abuse to evaluate their experiences away from the home environment and reflect on the self-impact of such experiences (Banyard and Cantor, 2004; Wright et al., 2009), and has been proposed as an important developmental task at this stage of life (Wright et al., 2009). Thus, in Study 3, based on retrospective recall, we assessed whether the age range during which adverse events occurred differentially predicted loneliness, and whether different social mechanisms linked trauma experiences with loneliness among young adults.

### Materials and methods

Participants (*N* = 566) were recruited through a university online research recruitment portal. Once again, participants were primarily female and white/Caucasian (see Table 1). Paper surveys were completed in person and participants were compensated with partial course credit. The same measures as in Study 2 were completed, including demographic information, loneliness (Cronbach’s α = 0.94) perceived social support (Cronbach’s α = 0.91), social connectedness (Cronbach’s α = 0.95), and unsupportive interactions with friends (Cronbach’s α = 0.93) and parents (Cronbach’s α = 0.93). When completing the Early Life Trauma Inventory (ELTI) (Bremner et al., 2007), participants indicated whether the events occurred during specified age ranges (0–5 years of age; 6–12 years of age; and 13–18 years of age). For each age range, participants indicated experiences of general trauma (Cronbach’s α = 0.94), physical punishment (Cronbach’s α = 0.89), emotional abuse (Cronbach’s α = 0.94), and sexual events (Cronbach’s α = 0.86). This study was approved by the Carleton University Research Ethics Board (#106215).

The statistical analyses followed the same approach as in the previous studies. The power to detect a medium effect size of partial *R*^2^ = 0.05 at *p* = 0.05 with the number of variables and sample size of the present study was β = 0.99. For none of the variables did the rate of missing responses exceed 1%.

### Results

As in Study 2, males reported significantly more experiences of physical punishment (*M* = 2.38, *SD* = 1.58) than females (*M* = 1.68, *SD* = 1.51), *F*(1,557) = 22.81, *p* < 0.001. Conversely, females reported significantly more experiences of unwanted sexual events (*M* = 1.20, *SD* = 1.62) than males (*M* = 0.49, *SD* = 0.88), *F*(1,556) = 25.34, *p* < 0.001. There were no significant gender differences in reported emotional abuse (*M* = 2.87, *SD* = 1.73) or general trauma (*M* = 3.58, *SD* = 1.99). Correlations among adverse experiences were all moderately positive, ranging from *r* = 0.19 (physical punishment and sexual events) to *r* = 0.39 (between physical punishment and emotional abuse). There was no gender difference in feelings of loneliness (*M* = 43.87, *SD* = 11.99). As seen in Table 1, almost a third of (31.6%) of the sample reported moderately high to high loneliness scores (50 or greater).

**FIGURE 3:**
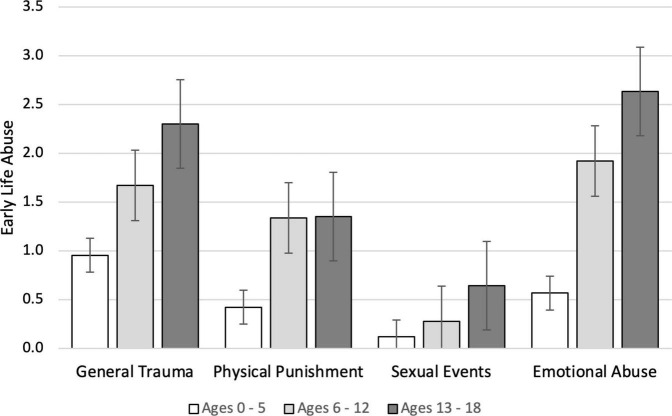
Mean frequency of early life abuse (±SE) as a function of type of abuse and the age range at which they were reported to have been experienced in Study 3. *N* = 566. Missing data < 1%.

As seen in Figure 3, reports all types of abuse increased in average frequency as age increased. Emotional abuse was the most frequently reported type of abuse at all ages, nearly double the frequency of reported physical punishment between ages 13 and 18.

A multiple linear regression analysis conducted to assess the relations between the experiences of early life trauma (collapsed across age of occurrence) and current loneliness indicated that taken together, early life traumas were associated with greater loneliness, *R*^2^ = 0.142, *F*(4,560) = 23.23 *p* < 0.001. Examination of the regression coefficients in Table 3 suggest that all forms of early life trauma and abuse were correlated with greater loneliness, but only emotional abuse and to a lesser degree unwanted sexual events contributed unique variance to loneliness.

**TABLE 3:** Linear regression coefficients predicting young adult loneliness based on early life adverse events prior to the age of 18 years in Study 3.

	*b*	*SE*	*B*	*r*
Physical punishment	0.03	0.33	0.004	0.14***
Sexual events	0.67	0.33	0.09*	0.17***
Emotional abuse	2.54	0.32	0.36***	0.37***
General trauma	–0.37	0.26	–0.06	0.10*

*p < 0.05; ***p < 0.001. *N* = 566.

#### Impact of the age during which abuse occurred

Multiple regression analyses assessed whether loneliness was differentially predicted by experiences of sexual events or emotional abuse (separate analyses; these were the only two dimensions of abuse related to loneliness) depending on the age at which the experiences were reported (0–5 years of age, 6–12 years of age, and 13–18 years of age). Encountering unwanted sexual events was mildly associated with loneliness, *R*^2^ = 0.025, *F*(3,559) = 4.84, *p* = 0.002, particularly when they were recalled to have occurred in middle to late childhood (Table 4). Experiences of emotional abuse across the age ranges were a strong predictor of loneliness, *R*^2^ = 0.157, *F*(3,559) = 34.59, *p* < 0.001, but as with sexual events, examination of the regression coefficients suggested that loneliness was increasingly predicted by abuse that occurred in more recent years.

**TABLE 4:** Linear regression coefficients predicting loneliness from reported childhood experiences of sexual events and emotional abuse at different age ranges in Study 3.

	*b*	*SE*	*B*	*r*
**Sexual events**				
Ages 0 – 5	0.50	0.92	0.03	0.08*
Ages 6 – 12	1.34	0.68	0.10*	0.13***
Ages 13 – 18	0.84	0.41	0.09*	0.12**
**Emotional abuse**				
Ages 0 – 5	0.82	0.46	0.08	0.26***
Ages 6 – 12	0.95	0.42	0.14*	0.35***
Ages 13 – 18	1.59	0.38	0.23***	0.37***

*p < 0.05; **p < 0.01; ***p < 0.001. *N* = 566.

To assess the mediating role of social processes on the link between early life sexual events and emotional abuse at various ages and loneliness, mediation analyses were conducted separately at each age range. As seen in Table 5, the patterns were relatively consistent across the age ranges and abuse types. However, the relations between childhood sexual events and loneliness were fully accounted for by social processes, and in particular perceptions of the availability of social support and feelings of social connection (but not unsupportive interactions with friends or parents). In contrast, with the exception of memories of emotional abuse at very early ages (0–5 years), emotional abuse continued to have implications for loneliness among young adults, beyond the role of social processes. This said, as in Study 2, at all three age ranges, the diminished feelings of social support and social connectedness along with unsupportive interactions with friends (but not parents) were significant mediators in the relations between childhood emotional abuse and loneliness.

**TABLE 5:** Mediation models of the relations between early life events and loneliness mediated by social factors across the three age ranges in Study 3.

	Total effect (*c*)	Direct effect (*c’*)	Indirect effects (*a_*i*_b_*i*_*)			
			Perceived social support	Social connectedness	Unsupportive interactions with friends	Unsupportive interactions with parents
** Sexual events**						
Ages 0 – 5	1.64* *SE* = 0.80	–0.23 *SE* = 0.38	0.41, *SE* = 0.23 CI_0.95_ [0.01, 0.91]	1.28, *SE* = 0.60 CI_0.95_ [0.15, 2.55]	0.13, *SE* = 0.12 CI_0.95_ [–0.10, 0.38]	0.05, *SE* = 0.05 CI_0.95_ [–0.04, 0.18]
Ages 6 – 12	1.85** *SE* = 0.59	–0.06 *SE* = 0.28	0.31, *SE* = 0.16 CI_0.95_ [0.02, 0.64]	1.43, SE = 0.42 CI_0.95_ [0.65, 2.29]	0.10, *SE* = 0.08 CI_0.95_ [–0.04, 0.28]	0.06, *SE* = 0.06 CI_0.95_ [–0.04, 0.19]
Ages 13 – 18	1.11** *SE* = 0.39	0.27 *SE* = 0.19	0.15, *SE* = 0.10 CI_0.95_ [–0.03, 0.35]	0.58, *SE* = 0.23 CI_0.95_ [0.14, 1.02]	0.07, *SE* = 0.05 CI_0.95_ [–0.01, 0.18]	0.04, *SE* = 0.04 CI_0.95_ [–0.04, 0.13]
** Emotional abuse**						
Ages 0 – 5	2.53*** *SE* = 0.40	–0.07 *SE* = 0.21	0.59, *SE* = 0.14 CI_0.95_ [0.34, 0.90]	1.61, *SE* = 0.32 CI_0.95_ [1.00, 2.25]	0.25, *SE* = 0.09 CI_0.95_ [0.11, 0.44]	0.14, *SE* = 0.11 CI_0.95_ [–0.07, 0.35]
Ages 6 – 12	2.38*** *SE* = 0.27	0.34* *SE* = 0.16	0.43, *SE* = 0.09 CI_0.95_ [0.27, 0.61]	1.36, *SE* = 0.20 CI_0.95_ [0.98, 1.75]	0.23, *SE* = 0.07 CI_0.95_ [0.10, 0.39]	0.03, *SE* = 0.09 CI_0.95_ [–0.16, 0.19]
Ages 13 – 18	2.55*** *SE* = 0.27	0.30^+^ *SE* = 0.16	0.45, *SE* = 0.08 CI_0.95_ [0.29, 0.61]	1.50, *SE* = 0.18 CI_0.95_ [1.16, 1.85]	0.26, *SE* = 0.08 CI_0.95_ [0.12, 0.43]	0.04, *SE* = 0.09 CI_0.95_ [–0.14, 0.22]

^+^*p* < 0.10; *p < 0.05; **p < 0.01; ***p < 0.001. *N* = 566.

### Discussion

The results of Study 3 replicated those of Study 2, in that emotional abuse predicted young adult loneliness, and this relationship was mediated by perceived social support, social connectedness and unsupportive interactions with friends, but not unsupportive interactions with parents. However, the age at which emotional abuse occurred had slightly different implications for the mediation model. Specifically, emotional abuse occurring between ages 0–5 did not uniquely predict young adult loneliness, whereas both emotional abuse between ages 6–12 and between ages 13–18 did. Retrospective recall may have had an impact on these results, particularly concerning the accuracy of memories occurring between ages 0–5. Nonetheless, our results are in line with previous research (Khan et al., 2015) suggesting that emotional abuse in later childhood and adolescence had stronger links with psychological distress. Similarly, it had been reported that childhood maltreatment that only occurred between ages 0–5 did not predict later symptoms of mental health problems (Russotti et al., 2021). These authors noted, however, that 12% of participants only experienced maltreatment between ages 0–5, whereas most children reported it throughout childhood and adolescence, and children who experienced maltreatment in both early childhood and in adolescence were at a higher risk for adverse mental health outcomes.

Our results suggest that young adults may be victims of emotional abuse more frequently than other forms of early life abuse or trauma, which aligns with previous research findings (Raissian et al., 2014; Grasso et al., 2016). However, emotional abuse was associated with more frequent physical punishment (in both Studies 2 and 3), which may account for why physical punishment was not a unique predictor of loneliness. In addition, the magnitude of the correlation between sexual events and loneliness was the same across the two studies, and thus may have emerged in Study 3 as a result of the increased power due to sample size differences. Indeed, the variance accounted for in such feelings was small, and the relationship was fully accounted for by diminished perceptions of social support and connection.

While perceived support, social connectedness and unsupportive interactions mediated the relationship between emotional abuse at all ages and subsequent loneliness in young adulthood, emotional abuse at the older ages contributed unique variance to loneliness levels after accounting for social processes. It appears that the mid and older childhood years represent a particularly vulnerable age for emotional abuse and its impacts on later loneliness, which is consistent with reports suggesting that emotional neglect that begins during the ages of 6–11 renders individuals especially vulnerable to poor outcomes (Khan et al., 2015; Schalinski et al., 2016).

## General discussion

Across three studies, it was apparent that loneliness is endemic among young people, with a quarter to a third of each sample reporting moderately high to severe levels of loneliness. While fundamental social processes, including perceptions of social support, social connectedness, and unsupportive interactions with friends were implicated in feelings of loneliness, so too were early life experiences of abuse, and in particular emotional abuse.

The relationship between adverse childhood experiences and mental health outcomes, including feelings of loneliness has previously been documented. However, given the retrospective correlational nature of most research, including the present study, the mechanisms by which childhood trauma promotes psychological distress and feelings of loneliness in young adulthood are poorly understood (Colburn et al., 2021). Study 1 confirmed that childhood abuse was uniquely associated with greater loneliness among young adults. Although other traumatic stressors were reported, including the loss of a loved one, witnessing something negative happening to someone else, or their own experiences of assault as adults, none accounted for unique variance in loneliness over and above experiences of physical or sexual abuse while growing up. This may suggest that the relationship between childhood experiences and outcomes in young adults is not simply a function of revictimization or the proliferation of stressor experiences.

Much of the current research into the effects of childhood abuse is dominated by studies investigating physical and sexual abuse (Stoltenborgh et al., 2015; Poole et al., 2018). Yet, Studies 2 and 3, which included consideration of early life emotional abuse, revealed this to be a powerful predictor of loneliness among young adults. Although other forms of child abuse tended to co-occur with emotional abuse, the latter was reported nearly twice as frequently as physical abuse and four times more frequently than sexual abuse. Our results add to the mounting evidence pointing to the alarming frequency and detrimental impacts of early life emotional abuse. One reason for the comparative lack of research into emotional abuse is the difficulty in defining it and capturing the experience (Baker, 2009; Tonmyr et al., 2011). Retrospective recall is likely biased by more recent experiences, although it did not seem in Study 3 that reports of emotional abuse in early childhood predominated, despite recollections of such abuse in mid to late childhood. Rather than resulting in a bias of over-reporting, it may be that later memories of these experiences are more vivid, and are more easily understood and interpreted as having constituted emotional abuse (Baker, 2009; Wright et al., 2009).

Childhood abuse has been associated with loneliness, which might, in part, stem from negative perceptions of social support, diminished social connectedness, and unsupportive peer interactions (Gibson and Hartshorne, 1996; Dodson and Beck, 2017; Aakvaag et al., 2019). It had been suggested that childhood abuse may promote a sense that social relationships are too risky and hence a disinclination to seek support or a propensity to communicate with others in a manner that elicits unsupportive reactions (Lee and Robbins, 1995; Lee et al., 2001; Williams and Galliher, 2006). While social support is an important factor in promoting resilience after experiences of childhood abuse (Leung et al., 2022) and protecting against feelings of loneliness (Riggio et al., 1993; Chen and Feeley, 2014), gaps remain in the understanding of critical elements of social support implicated in loneliness among young adults (Leung et al., 2022).

In Study 1, the relationship between experiences of childhood abuse and loneliness was partially accounted for by perceptions that peers and parents were not available as sources of support (although neither accounted for unique variance over the other). This relationship to lower perceived social support (across sources) was replicated in regard to sexual abuse (Study 3) and emotional abuse (Studies 2 and 3). However, a cogent aspect of young people’s social experiences that was disrupted by childhood abuse (at any age) was a sense of social connection. This finding was congruent with research suggesting that the broader cognitive schemas related to social connectedness may play an important role in loneliness (Gibson and Hartshorne, 1996; Dodson and Beck, 2017), and has been proposed as a fundamental mechanism linking emotional abuse and psychological distress and mental illness later in life (Wright et al., 2009). Low social connectedness may be tied to maladaptive social cognitions that were shaped by experiences of early life emotional abuse, contributing to the development of schemas of vulnerability to harm, personal shame, and a lack of mattering to others (Wright et al., 2009; Flett et al., 2016). How children appraise emotional abuse and integrate it into their sense of self and view of social relationships may be especially important in predicting psychological outcomes (Wright et al., 2009).

Early life emotional abuse was associated with reports of frequent unsupportive interactions with friends and parents, although only the interactions with friends were associated with greater loneliness. Social support from friends in the adolescent and young adult stage of life may have a greater impact on mental health and well-being than family support (Secor et al., 2017; Von Soest et al., 2020). It is possible that childhood emotional abuse, which is most often inflicted by family, limits the social skills of children to form healthy peer relationships (Fergusson and Horwood, 1999; Gayman et al., 2011; Aakvaag et al., 2019) and they are more likely to experience social rejection (Lev-Wiesel and Sternberg, 2012; Li et al., 2022). Much like the experience of rejection, unsupportive reactions from parents may lead to the development of relationships with deviant peer groups (Fergusson and Horwood, 1999; Li et al., 2022) who may be less likely to provide positive social support. Moreover, early life emotional abuse has been strongly linked to revictimization later in life (Lev-Wiesel and Sternberg, 2012; Gama et al., 2021), and this may be in the form of unsupportive interactions with emotionally abusive friends. Finally, childhood emotional abuse may influence how an individual perceives not only the availability of social support, but also its quality (Williams and Galliher, 2006). In essence, they may perceive friends as unsupportive regardless of their actual behavior.

## Limitations and conclusion

Meaningful and supportive relationships based on secure attachment and mutual reciprocity of support have been described as critical human needs, much like food and water are essential biological needs (Tomova et al., 2020, 2021). In line with this, it has been suggested that feelings of loneliness may have evolutionary significance, in that they motivate an individual to seek connection, which promotes the survival of the individual and the group (Baumeister and Leary, 1995). It may be that early life emotional abuse that is associated with diminished need for social connection similarly serves in an adaptive capacity, protecting harmed individuals from further exposure to destructive relationships. Indeed, while trauma is often considered as a cogent factor that fosters psychopathology, depending on the psychosocial context and biopsychosocial factors, these experiences can promote resilience (Ungar, 2021). Such protective outcomes were not evaluated in the present study and would likely take some time to emerge. Indeed, before drawing conclusions from the retrospective self-reports and the correlational design of the present study for interventions to alleviate loneliness (and other mental health outcomes), it is important to understand the significance of the social mechanisms that appear to be implicated. While they may be dysfunctional among some populations, for others they may be protective. A limitation of the present studies was that the samples all comprised self-selected university students, who arguably given their immersion in a highly peer-involved social environment, may represent a more socially functional population. At the same time, students are being increasingly recognized as a population at high risk for loneliness and the associated mental health concerns (Diehl et al., 2018; Hysing et al., 2020). Indeed, while the present investigation was not conducted during the COVID-19 pandemic, it became apparent that university students were substantially affected by the social isolation that occurred due to pandemic restrictions, and understanding the mechanisms associated with loneliness among student populations is meaningful (Bu et al., 2020).

Given the limited social connectedness and interpersonal schemas regarding shame and personal safety that arises in relation to experiences of emotional abuse, social surrogates (e.g., fictional characters, pets, or video games) may better serve to meet relational needs, at least temporarily (McConnell et al., 2011; Gabriel et al., 2017; Vella et al., 2019; Paravati et al., 2021). Targeted efforts to enhance social connections and recognition from others may be another fruitful strategy (Haslam et al., 2016; Wang et al., 2022). For example, an intervention that builds on the important role that meaningful social groups play in people’s lives (Groups4Health) may serve as an effective strategy for reducing loneliness and promoting wellness (Cruwys et al., 2022). Bringing people together to form a common identity can help furnish them with the confidence and skills they need to engage in ways that are self-affirming. Such strategies might well be more sustainable than individualized clinical interventions, as they fortify social connections in individuals’ day-to-day lives (Haslam et al., 2016; Cruwys et al., 2022). While an understanding was gained of the cluster of social processes that emerge from childhood abuse that appear to create links to diminished well-being of young adults, assessments of causal and functional relations are still much needed. Such studies may require multi-method prospective designs that further take gender, socioeconomic status and ethnocultural factors into consideration. Nonetheless, across three studies, the present investigation provides consistent evidence that early life abusive experiences are associated with loneliness among young adults, and these relations are likely determined by psychosocial processes that may develop in response to such abuse.

## Data availability statement

The raw data supporting the conclusions of this article will be made available by the authors, without undue reservation.

## Ethics statement

The studies involving human participants were reviewed and approved by Carleton University Research Ethics Board. The patients/participants provided their written informed consent to participate in this study.

## Author contributions

JL and KM wrote the initial drafts of the manuscript and were involved in the conception and analyses of the studies. AA and CC were involved in the conception and conduct of the studies. HA contributed to the conception of the research and writing of the manuscript. KM and HA acquired the funding to support the research. All authors contributed to the article and approved the submitted version.
